# Analysis of Inflammatory Mediators in Type 2 Diabetes Patients

**DOI:** 10.1155/2013/976810

**Published:** 2013-05-20

**Authors:** Ahmed Al-Shukaili, Saif AL-Ghafri, Safia Al-Marhoobi, Said Al-Abri, Jawad Al-Lawati, Masoud Al-Maskari

**Affiliations:** ^1^Health & Social Development Sector, The Research Council of Oman, P.O. Box 1422, 130 Muscat, Oman; ^2^Emergency Departments, Sultan Qaboos University Hospital (SQUH), P.O. Box 35, 123 Muscat, Oman; ^3^Studies & Planning, Statistics Section, The Research Council, P.O. Box 1422, 130 Muscat, Oman; ^4^Public Health, Armed Forces Medical Services Headquarters, Ministry of Defense, P.O. Box 928, 13 Muscat, Oman; ^5^Department of Non-Communicable Diseases Surveillance and Control, Ministry of Health, P.O. Box 393, 100 Muscat, Oman

## Abstract

The main aim of this study is to assess the inflammatory markers in type 2 diabetes mellitus (T2DM) by measuring some cytokines concentrations and lymphocytes subset and correlate them with other laboratory investigations. Fifty-seven patients with type-2 diabetes and 30 healthy volunteers were enrolled in this study. Data for the C-reactive protein (CRP), haemoglobin, HbA1c, and autoantibody levels were obtained from the patients files. The cytokine concentrations were measured in patient's serum using commercially available ELISA assays. Lymphocytes subsets were measured by flow cytometric methods. The levels of IL-1*β*, IL-6, IL-15, and TNF-*α* were found to be decreased in T2DM patients, whereas the levels of IL-10, IFN-*γ*, and caspase-1 were increased, compared to normal controls. T2DM patients with hypertension show significantly decreased levels of IL-1*β* and caspase-1 compared to patients without hypertension. No significant differences in lymphocytes subset between cases and normal control were observed. Significant correlations were found between HbA1c and IL-6; body mass index (BMI) was significantly correlated with CRP, TNF-*α*, and phosphate; the weight (Wt) was associated with CRP and IFN-*γ*. In conclusion, an alteration in the function of the immune system was observed in T2DM patient.

## 1. Introduction

Type 2 diabetes mellitus (T2DM) represents a significant global health problem. The burden of diabetes has increased sharply in Oman over the last decade, rising from 8.3% in 1991 to 11.6% in 2000, and 12.3% in 2008 among adults aged 20 years and older [1, 2].

Inflammation is considered to be a key regulator of the pathogenesis of T2DM, but what triggers this inflammation still unknown [3]. However, it may be related to obesity. Obesity is associated with enlargement of adipose tissue and consequently increases the number of adipose tissue macrophages [4, 5]. These macrophages are responsible for almost all adipose tissue tumor necrosis factor-*α* (TNF-*α*) expression, significant amounts of interleukin-6 (IL-6), and other acute-phase response markers and mediators of inflammation [5–7]. 

Many proinflammatory cytokines play a central role in inflammatory reaction and were shown to increase the risk of T2DM [8, 9]. These pro-Inflammatory cytokines can enhance insulin resistance directly in adipocytes, muscle and hepatic cells, leading to systemic disruption of insulin sensitivity and impaired glucose homeostasis. Increased levels of these pro-inflammatory cytokines lead to hepatic production and secretion of acute-phase proteins such as C-reactive protein (CRP), plasminogen activator inhibitor-1 (PAI-1), amyloid-A, *α*1-acid glycoprotein, and haptoglobin. These proteins appear in the early stages of T2DM, and their circulating concentrations increase as the disease progresses [3, 8–10]. 

It has been reported that normal individuals with detectable levels of IL-1*β* and elevated levels of IL-6 had an independently increased risk to develop T2DM, whereas those with increased concentrations of IL-6 but undetectable levels of IL-1*β* had no significantly increased risk [11, 12]. Another study showed that levels of IL-6, TNF-*α*, and TNF-receptor were elevated in insulin-treated, but not in sulfonylurea-treated patients [13]. Moreover, levels of serum glucose, pro-inflammatory cytokines (IL-6, IL-12, and TNF-*α*), endothelial dysfunction markers (vascular cell adhesion molecule-1 (VCAM-1), intercellular adhesion molecule-1 (ICAM-1), and nitric oxide), and lipid abnormality were highest in T2DM with cardiovascular complications [14]. Recently, it has been shown that T2DM patients had a significantly higher CD14 (+) CD16 (+) fluorescence intensity, TLR4 expression, and serum IL-6 and C-reactive protein (CRP) levels, compared to normal controls [15].

Here in this study, we extend the analysis of the role of inflammation in T2DM by measuring the levels of several cytokines, including IL-1, IL-6, IL-10, IL-15, IFN-*γ*, TNF-*α*, and caspase-1 in T2DM and correlate them with other laboratory investigations. We will also examine whether there is an alteration in lymphocytes subsets in T2DM compared to the normal controls.

## 2. Patients and Methods

Fifty-seven patients (28 male, and 29 females, mean age 52 ± 11.5) with type 2 diabetes and 30 healthy volunteers (20 male, and 10 females, mean age 35 ± 7) were enrolled in this study (Table 1). Patients attended the outpatients clinic at Sultan Qaboos University Hospital (SQUH), Muscat, Oman. Confirmed T2DM patients were selected randomly; however, cases associated with other inflammatory diseases such as cancer or autoimmunity were excluded from this study. Informed consent was obtained from each subject. The Medical Research and Ethics Committee (MREC) at the College of Medicine, Sultan Qaboos University (SQU), approved this study. 

Data for the C-reactive protein (CRP), hemoglobin, HbA1c and autoantibody levels were obtained from the patients files. The cytokine concentrations were measured in patients sera using commercially available ELISA assays (R&D Systems), performed according to the manufacturer's instructions. The lymphocytes subset was measured using monoclonal antibodies available as a Multiset panel (BD, USA, and analyzed using FACSCalibur flow cytometry (BD, USA), equipped with automated program.

### 2.1. Statistical Analysis

Statistical analyses were performed using SPSS software (20.0 version). Data normality was tested using KS test. Quantitative data were presented as mean ± SD. The statistical significance between means was estimated by Student's *t*-test (independent samples) when appropriate. Pearson's correlation coefficient (*r*) was used to measure the strength of the association between the two variables. Differences were considered statistically significant at *P* < 0.05.

## 3. The Results

### 3.1. Cytokines Levels

**Figure 1:**
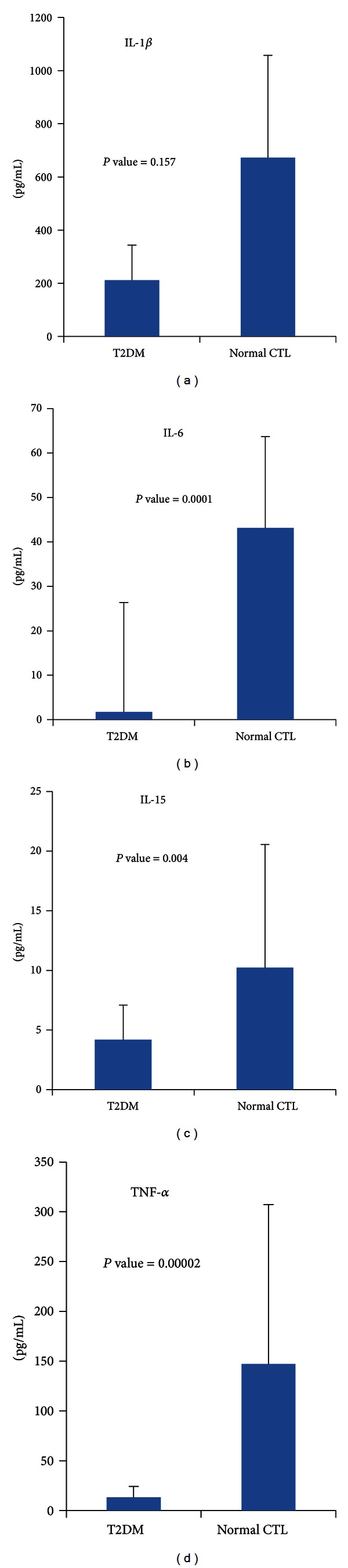
Decreased cytokine profile in T2DM.

The levels of IL-1*β*, IL-6, and IL-15 TNF-*α* were decreased in T2DM patients compared to normal controls (Figure 1). Mean baseline level of IL-1 (211 ± 132 pg/mL) was lower among T2DM compared with control subjects (672 ± 385 pg/mL) but statistically insignificant (*P* value = 0.157).

However, the mean value of IL-6 was significantly (*P* value of 0.0001) lower in T2DM cases (1.76 ± 2.5 pg/mL), compared to control subjects (43 ± 20 pg/mL). The mean value of IL-15 was 4 ± 2.9 pg/mL, which is also significantly lower (*P* value = 0.004) than the mean value for the controls (10.2 ± 10.3 pg/mL). Similar significant difference (*P* value = 0.00002) was observed for TNF-*α*; the mean baseline levels were 13 ± 11 pg/mL for T2DM patients and 147 ± 160 pg/mL for the control subjects.

When the patients' data was sorted according the treatment (insulin or sulfonylurea), no significant difference was observed between those patients treated with insulin only or those patients treated with sulfonylurea (data not shown).

**Figure 2:**
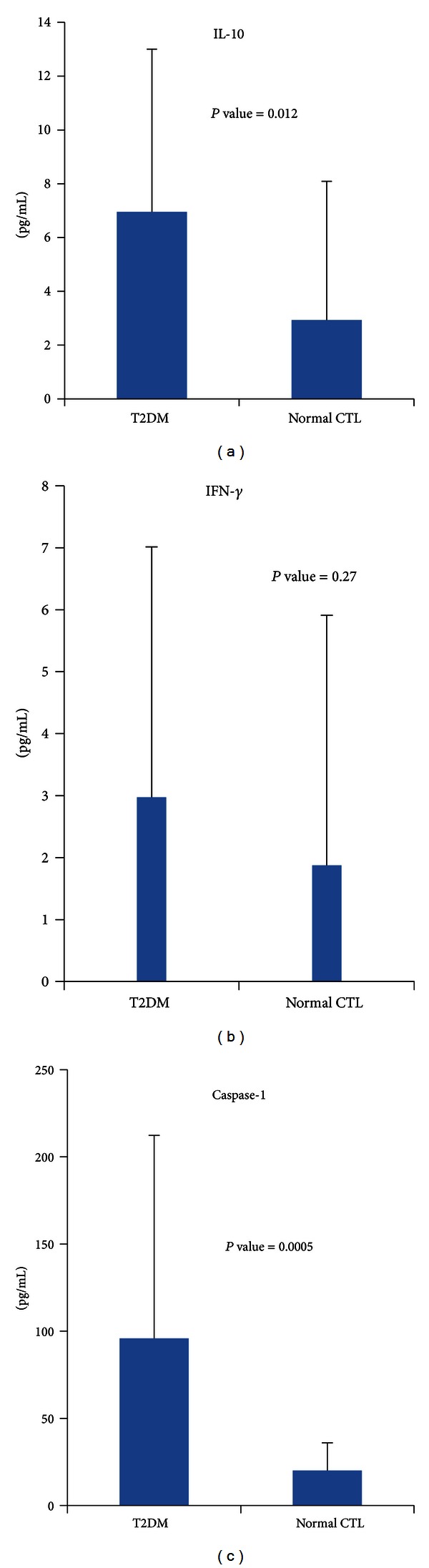
Increased cytokine profile in T2DM.

The levels of IL-10, IFN-*γ*, and caspase-1 were increased in T2DM patients compared to normal controls (Figure 2). Mean baseline levels of IL-10 (6.95 ± 6 pg/mL) were significantly higher (*P* value = 0.012) among T2DM patients compared to control subjects (2.9 ± 5.15 pg/mL). Likewise, the mean value of caspase-1 was 95.9 ± 116 pg/mL, which is significantly higher (*P* value = 0.0005) than the mean value for the control (20.2 ± 15 pg/mL). However, no significant differences were observed in the levels of IFN-*γ* between patients (2.97 ± 4 pg/mL) and controls (1.8 ± 4.1 pg/mL).

When the patient's data was distributed according to the treatment, no significant difference was observed between patients in insulin only or patients treated with sulfonylurea; this is obviously because of the very small number in both groups; a large cohort should be used in the future.

### 3.2. Effect of Hypertension on Cytokine Production

**Figure 3:**
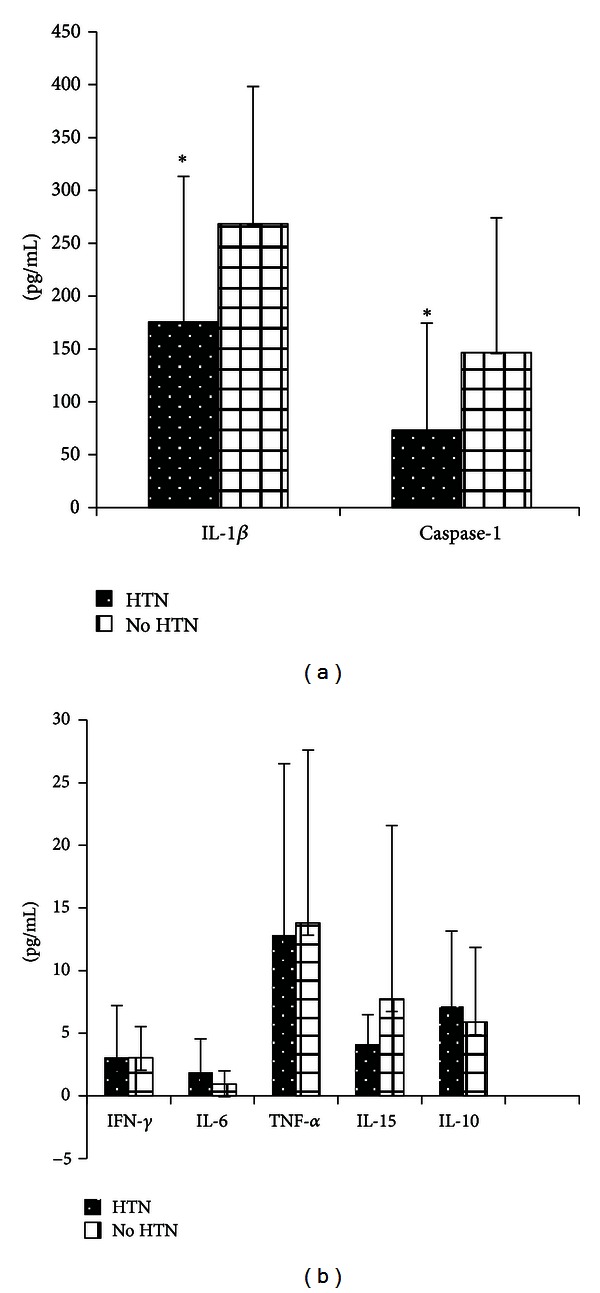
Cytokine profile in T2DM with or without hypertension. *Significant difference (*P* value < 0.05).

T2DM Patients with hypertension show decreased levels of pro-inflammatory cytokines (Figure 3). Interestingly; T2DM patients with hypertension shows significantly decreased levels of IL-1*β* and caspase-1 (*P* value = 0.024, 0.028, resp.). TNF-*α* and IL-15 levels were also decreased but statistically insignificant. IL-6 and IL-10 levels were slightly increased in T2DM patients with hypertension.

### 3.3. Lymphocyte Subsets

Lymphocytes subsets in T2DM and normal controls are shown in Table 2; several immune cells were measured, including lymphocytes (CD3+), T helper cells (CD4+), T-cytotoxic cells (CD8+), double positive CD4/CD8, natural killer cells (CD56+), and B cells (CD19+), and no significant differences between T2DM patients and normal controls were observed, Table 2.

### 3.4. Correlation Analysis

HbA1c was positively correlated with IL-6 with a significant *P*-value of 0.005, and BMI was positively and significantly correlated with CRP (*P* value = 0.001), TNF-*α* (*P* value = 0.013) and phosphate (*P* value = 0.008). The weight (Wt.) of the patients was significantly correlated with CRP and IFN-*γ*, *P* values of 0.11 and 0.044, respectively. In addition, calcium level was correlated with phosphate (*P* value = 0.004), hypertension (*P* value = 0.032), LDL (*P* value = 0.03) and dyslipidemia (*P* value = 0.011), Table 3.

## 4. Discussion 

Our data showed decreased levels of IL-1*β*, IL-6, IL-15, and TNF-*α* and increased levels of IL-10, IFN-*γ*, and caspase-1 in T2DM compared to healthy controls. Decreased levels of inflammatory cytokines in our study were in disagreement with previous findings. A recent study by Marques-Vidal et al. (2013) found that subjects with T2DM had increased levels of IL-6, TNF-*α*, and hs-CRP, while no association was found with IL-1*β* [16]. Moreover, it was reported that, high levels of inflammatory cytokines appear in early stage of T2DM and capable of predicting the development of type 2 diabetes through diminishing insulin sensitivity [9, 10]. This discrepancy can be attributed to the (I) duration of the diseases; the majority of patients included in our study have a long disease duration (greater than 5 years), (II) small sample size, and (III) the differences in age and sex of the studied groups; the age of the normal controls was lower than that of T2DM patients. Moreover, there were only 10 females on controls versus 29 females on T2DM group. These factors may have played essential roles in the cytokine production among these two study groups. 

Mavridis et al. investigated inflammatory cytokines in insulin-treated T2DM patients and showed increased levels of IL-6, TNF-*α* in insulin-treated T2DM patients compared to sulfonylurea-treated patients. Also, they found a positive association between waist circumference and IL-6 and a significant correlation between HbA1c and IL-6 [13]. When we sorted our data of cytokines levels, according to the treatment (insulin or sulfonylurea) of the patients, we did not find any significant differences; this may be due to a small sample size in our study. However, our results showed that HbA1c levels were correlated with IL-6 levels, which is in accordance with previous findings [11, 12]. Nevertheless, from all these data, we cannot conclude whether poor glycaemic control leads to inflammation or whether inflammation leads to higher glucose levels; further studies are needed to assess such questions. 

Moreover, our study showed that BMI was positively correlated with CRP, TNF-*α*, and phosphate levels; and the weight was positively correlated with CRP and IFN-*γ*, which is in accordance with previous findings [16–18]. This correlation can be explained as follows: obesity is associated with enlargement of adipose tissue and consequently increases the number of adipose tissues macrophages [4–6]. These macrophages are responsible for almost all adipose tissue TNF-*α* expressions and other acute-phase response markers and mediators of inflammation [5–7]. TNF-*α*, secreted by adipose tissue, may play a critical role in insulin resistance and the pathogenesis of type 2 diabetes. Several studies indicated that increased levels of cytokines and acute-phase proteins can participate in maintaining the insulin-resistant state [16, 19].

In T2DM patients with hypertension, the pathophysiology of cardiovascular disease is multifactorial; for example angiotensin II may be to a large degree responsible for triggering vascular inflammation by inducing oxidative stress [20]. However, our data shows that T2DM patients with hypertension had significantly lower levels of IL-*β* and Caspase-1 and slightly higher levels of IL-6 and IL-10, compared to patients with no hypertension. This can be attributed to the treatment of hypertension, such that drugs that target the renin-angiotensin system may reduce blood pressure and inflammation in T2DM patients with hypertension [20]. 

In conclusion, we found that, patients with established T2DM, had different cytokine profile than healthy controls, without a significant change in lymphocyte subsets; this indicates that, there is an alteration in the function of the immune system in T2DM patient.

## Figures and Tables

**Table 1:** Demographic information and laboratory investigations of the T2DM patients.

Characteristics	Mean ± SD/*n* (%)	
	T2DM patients	Healthy controls
Age	52.3 ± 11.5	35 ± 7
Sex		
Male	28 (49)	20 (67%)
Female	29 (51)	10 (33%)
Disease duration		
≥5 years	51 (89.4)	NA
<5 years	6 (10.6)	
Height	158.8 ± 7.8	165 ± 10
Weight	76.8 ± 13.8	73 ± 10.2
BMI	30.4 ± 5.2	NA
HbA1c	8.2 ± 1.4	NA
CRP	11.1 ± 24.6	NA
Vitamin D	44.2 ± 20.2	NA
Hypertension	38 (67)	0
TC	5.1 ± 1.1	NA
LDL	3.1 ± 1.0	NA
HDL	1.2 ± 0.4	NA
TG	1.6 ± 1.1	NA
Current smokers	2 (3.5)	0
Treatment (%)		NA
Insulin	12 (21)	
Oral		
(Metformin/sulfonylurea)	27 (47)	
(Metformin only)	4 (7)	
Oral + insulin	9 (16)	
Diet + exercise	5 (9)	

T2DM: type 2 diabetes mellitus; BMI: body mass index; HbA1c: glycated haemoglobin; CRP: C-reactive protein; TC: total cholesterol; LDL: low-density lipoprotein; HDL: high-density lipoprotein; TG: triglycerides. NA: not available.

**Table 2:** Lymphocytes subset in T2DM and normal controls.

	CD3+	CD8+	CD4+	CD4+/CD8+	CD56+	CD19+
Normal control, *n* = 30	69 ± 8.14	25 ± 5	38 ± 8	0.7 ± 1	12 ± 5	14 ± 4.7
T2DM, *n* = 59	71 ± 8.9	27 ± 8.2	43 ± 7.5	0.75 ± 1	14 ± 7.5	13 ± 4.7
*P* value	NS	NS	NS	NS	NS	NS

NS: not significant.

**Table 3:** Correlation analysis; data shows significant *P* value.

	IL-6	CRP	IFN-*γ*	TNF-*α*	Phosphate	HTN	LDL	Dyslipidemia
HbA1c	0.005	—	—	—	—	—	—	—
BMI	—	0.001	—	0.013	0.008	—	—	—
Wt.	—	0.011	0.044	—	—	—	—	—
Calcium	—	—	—	—	0.004	0.032	0.030	0.011

HbA1c: glycated haemoglobin; BMI: body mass index, Wt.: weight; CRP: C-reactive protein, HbA1c: glycated haemoglobin; TG: triglycerides; LDL: low-density lipoprotein; HTN: hypertension; TNF-*α*: tumor necrosis factor; IL-6: interleukin-6; IFN-*γ*: interferon.
